# The link between independent acquisition of intracellular gamma-endosymbionts and concerted evolution in *Tremblaya princeps*

**DOI:** 10.3389/fmicb.2015.00642

**Published:** 2015-06-25

**Authors:** Sergio López-Madrigal, Amparo Latorre, Andrés Moya, Rosario Gil

**Affiliations:** ^1^Institut Cavanilles de Biodiversitat i Biologia Evolutiva, Universitat de ValènciaValència, Spain; ^2^Área de Genómica y Salud de la Fundación para el Fomento de la Investigación Sanitaria y Biomédica de la Comunitat Valenciana (FISABIO) – Salud PúblicaValència, Spain

**Keywords:** mealybugs, nested endosymbiosis, homologous recombination, concerted evolution

## Abstract

Many insect species establish mutualistic symbiosis with intracellular bacteria that complement their unbalanced diets. The betaproteobacterium “*Candidatus* Tremblaya” maintains an ancient symbiosis with mealybugs (Hemiptera: Pseudococcidae), which are classified in subfamilies Phenacoccinae and Pseudococcinae. Most Phenacoccinae mealybugs have “*Candidatus* Tremblaya phenacola” as their unique endosymbiont, while most Pseudococcinae mealybugs show a nested symbiosis (a bacterial symbiont placed inside another one) where every “*Candidatus* Tremblaya princeps” cell harbors several cells of a gammaproteobacterium. Genomic characterization of the endosymbiotic consortium from *Planococcus citri*, composed by “*Ca*. Tremblaya princeps” and “*Candidatus* Moranella endobia,” unveiled several atypical features of the former's genome, including the concerted evolution of paralogous loci. Its comparison with the genome of “*Ca*. Tremblaya phenacola” PAVE, single endosymbiont of *Phenacoccus avenae*, suggests that the atypical reductive evolution of “*Ca*. Tremblaya princeps” could be linked to the acquisition of “*Ca*. Moranella endobia,” which possess an almost complete set of genes encoding proteins involved in homologous recombination. In order to test this hypothesis, we performed comparative genomics between “*Ca*. Tremblaya phenacola” and “*Ca*. Tremblaya princeps” and searched for the co-occurrence of concerted evolution and homologous recombination genes in endosymbiotic consortia from four unexplored mealybug species, *Dysmicoccus boninsis*, *Planococcus ficus*, *Pseudococcus longispinus*, and *Pseudococcus viburni*. Our results support a link between concerted evolution and nested endosymbiosis.

## Introduction

The advances in genome sequencing and the development of metagenomic methods have been critical for our knowledge of the bacterial world. Now that complete genomes from closely related species or even from different strains of the same species are available, numerous studies have focused on the diversity of gene repertoire and genome rearrangements (Abby and Daubin, 2007). Horizontal gene transfer (HGT), transposition and intragenomic recombination are known to be important sources of evolutionary novelties, being responsible for bacterial huge metabolic diversity and adaptive potential, which are remarkable among free-living bacteria (Casjens, 1998; Rocha, 2008). However, the analysis of bacteria that have acquired an intracellular host-dependent life-style revealed important constrains to these evolutionary mechanisms.

During the last 15 years, the complete genomes of many endosymbionts (i.e., obligate symbiotic bacteria that live inside eukaryotic cells) have become available. The best studied cases of endosymbiosis involve mutualistic associations with insects. Comparative genomics has allowed the identification of several commonalities among them, which are related with the stage of integration of the bacteria with their respective hosts (Moya et al., 2008; McCutcheon and Moran, 2012). Generally, intracellular bacteria have smaller genomes than their free-living relatives, mostly due to a reduction in their gene content (McCutcheon and Moran, 2012). Gene losses affect loci performing functions that are unnecessary in an intracellular environment or that can be provided by the host. Thus, highly reduced genomes (i.e., those from endosymbionts that have maintained a long relationship with their hosts) have typically lost most genes involved in DNA recombination and repair, present almost no gene duplications, lack transposable elements and prophages and present high levels of structural stability.

Many insects maintain obligate mutualistic symbiosis with more than one bacterial species, so that two evolutionary outcomes are possible: complementation through the establishment of a bacterial consortium or replacement of one endosymbiont by another (Moya et al., 2009). Mealybugs (Hemiptera: Pseudococcidae) are phloem-feeding insects that have been classified in subfamilies Phenacoccinae and Pseudococcinae (Hardy et al., 2008), and present an intricate variety of endosymbiotic relationships. Based on phylogenetic analysis, it has been suggested that a betaproteobacterial ancestor of “*Ca*. Tremblaya” infected a mealybug ancestor before the split of the two subfamilies. In subfamily Phenacoccinae, “*Ca*. Tremblaya phenacola” is the obligate endosymbiont in most tested mealybug species, excluding the tribe *Rhizoecini* and the genus *Rastrococcus*, where it has been replaced by different *Bacteroidetes* (Gruwell et al., 2010; Husnik et al., 2013). In subfamily Pseudococcinae, the obligate endosymbiont “*Ca*. Tremblaya princeps” has been classified in up to six different clusters (A–F) (Thao et al., 2002). Except for the *Ferrisia* and *Maconellicoccus* clusters (B and F, respectively), where no additional endosymbiont has been reported, “*Ca*. Tremblaya princeps” has been recurrently infected by different gammaproteobacteria, establishing nested endosymbiotic consortia in which each “*Ca*. Tremblaya princeps” cell contains several cells of the corresponding gammaproteobacterium (von Dohlen et al., 2001; Thao et al., 2002; McCutcheon and von Dohlen, 2011; Gatehouse et al., 2012; Koga et al., 2013).

Early approaches to the genomic characterization of “*Ca*. Tremblaya princeps” revealed several atypical features for an obligate endosymbiont, including the presence of a 5.7-kb duplicated fragment involving the complete ribosomal operon and its closest genomic context (Baumann et al., 2002). Paralogous loci were detected in several strains from a diverse set of Pseudococcinae mealybug species, indicating that the duplication event occurred at early stages of “*Ca*. Tremblaya princeps” diversification. In spite of this, paralogous fragments have remained identical within each strain genome, suggesting that they have been affected by concerted evolution (Baumann et al., 2002). Concerted evolution is a molecular process driven by DNA recombination mechanisms that leads to homogenization of duplicated loci within a species. Consequently, paralogous loci are more closely related to each other than to the corresponding orthologous regions in another species, even though the duplication event preceded the speciation event (Liao, 1999).

The endosymbiotic system identified in *Planococcus citri* (cluster E), where “*Ca*. Tremblaya princeps” harbors “*Ca*. Moranella endobia,” has been extensively studied (von Dohlen et al., 2001; López-Madrigal et al., 2011, 2013a,b; McCutcheon and von Dohlen, 2011; Husnik et al., 2013). The complete sequencing of the 138.9-kb genome of “*Ca*. Tremblaya princeps” from *P. citri* confirmed the presence of the identical duplicated loci although no DNA repair and recombination genes were detected (López-Madrigal et al., 2011; McCutcheon and von Dohlen, 2011). In contrast, its gammaproteobacterial partner “*Ca*. Moranella endobia” (with a 538.2-kb genome) still retains a diverse set of genes involved in both the RecF and RecBCD recombination pathways, the two redundant mechanisms for this function that are nearly ubiquitous in free-living bacterial species (Rocha et al., 2005; Spies and Kowalczykowski, 2005; López-Madrigal et al., 2013a). Recent sequencing of the genome of “*Ca*. Tremblaya phenacola” PAVE, the sole obligatory endosymbiont of the mealybug *Phenacoccus avenae*, revealed that it also possess a tiny genome (171.5 kb), suggesting that a severe gene loss must have affected the common ancestor of both “*Ca*. Tremblaya” species at the beginning of the obligate intracellular symbiosis (Husnik et al., 2013). The genome of “*Ca*. Tremblaya princeps” is an almost perfect subset of that from “*Ca*. Tremblaya phenacola,” which has retained many essential genes involved in metabolic and informational functions that are absent in “*Ca*. Tremblaya princeps” and must be provided by its nested endosymbiont “*Ca*. Moranella endobia” (Husnik et al., 2013). However, “*Ca*. Tremblaya phenacola” PAVE also lacks all genes involved in DNA recombination. Therefore, the maintenance of homologous recombination (HR) pathways in “*Ca*. Moranella endobia” could be at the root of the concerted evolution noticed in the “*Ca*. Tremblaya princeps” genome. If this hypothesis is correct, we expect to find HR-related genes and signals of recent concerted evolution in additional endosymbiotic consortia from Pseudococcinae mealybugs.

We have checked for the co-occurrence of both features by analyzing the nested endosymbiotic systems from four unexplored mealybug species. The gray sugarcane mealybug *Dysmicoccus boninsis*, the long tailed mealybug *Pseudococcus longispinus*, and the obscure mealybug *Pseudococcus viburni* are phylogenetically distant members of the tribe Pseudococcini (Hardy et al., 2008), and their gammaproteobacterial endosymbionts have been independently acquired (López-Madrigal et al., 2014). The vine mealybug *Planococcus ficus* is a close relative of *P. citri*. Additionally, we explored the origin of the ribosomal operon duplication in “*Ca*. Tremblaya” and analyzed the susceptibility of both “*Ca*. Tremblaya princeps” and “*Ca*. Moranella endobia” to HR. Our results support a link between concerted evolution and nested endosymbiosis, suggesting a great impact of the gamma-endosymbionts on the reductive evolution of “*Ca*. Tremblaya princeps” genome, not only at the functional but also at the structural level.

## Materials and methods

### Insect sample collection and DNA extraction

Insects belonging to the species *P. longispinus, P. viburni*, and *D. boninsis* were field collected in the Botanical Garden of the Universitat de València (València, Spain. 39° 28′ 11.667″ N, 0° 22′ 34.637 W), with permission from the curator of the garden, Dr. Jaime Güemes. *P. ficus* was sampled from a population reared on *Vitis vinifera* at the Mediterranean Agroforestal Institute, Universitat Politècnica de València (València, Spain. 39° 29′ 1.699 N, 0° 20′ 28.978″ W). This study did not involve endangered or protected species. Insects were stored in absolute ethanol at −20°C. Total insect DNA (_*T*_DNA) was extracted from adult female insects, where endosymbiont populations are expected to reach a peak (Kono et al., 2008), using JETFLEX Genomic DNA Purification Kit (GENOMED).

### DNA amplification and sequencing

PCR amplifications were performed on insect _*T*_DNA with appropriate primer pairs (see below), using 50–60 μmoles of each primer per 50 μl reaction, and the KAPATaq DNA Polymerase Kit (Kapa Biosystems). *P. citri*_*T*_DNA was used as a positive control. The thermal cycling protocol was as follows: an initial denaturation at 95°C for 5 min, followed by 35 cycles of 50 s at 95°C, 40 s at 55°C (or 52°C when indicated), and 2 min at 72°C, plus a final extension step of 7 min at 72°C. Amplicons were ABI sequenced at the sequencing facility of the Universitat de València.

Sequencing reads were quality surveyed and assembled with Staden Package (http://staden.sourceforge.net; Staden et al., 2000). Artemis software was used for sequence data management (http://www.sanger.ac.uk/resources/software/artemis/; Rutherford et al., 2000).

### Molecular and evolutionary analysis

“*Ca*. Tremblaya princeps” genomic fragments *leuA*-*rrs*1 and *prs*-*rrs*2 were PCR amplified with proper combinations of the already described primers leuA, prs5/6 and U16S (Baumann et al., 2002). The same primers, as well as OR-leuAR2 (5′-TCAGTMATTAHGGCWACCTGCAC-3′), OR-prsR2 (5′-AATAGCYAAGCGGGTCAAGGC-3′) and OR-UF2 (5′-TGGCGCATGCTGTATGAGTTC-3′), were used to sequence the PCR products. tRNAscan-SE (http://lowelab.ucsc.edu/tRNAscan-SE/; Lowe and Eddy, 1997) and ARAGORN (http://mbio-serv2.mbioekol.lu.se/ARAGORN/; Laslett and Canback, 2004) were used for the prediction of tRNA genes. All other genes were annotated by BLAST searches (http://blast.ncbi.nlm.nih.gov/Blast.cgi/; Altschul et al., 1997). The newly obtained sequences have been deposited in the GenBank database (*D. boninsis*, KF591104 and KF591105; *P. longispinus*, KF591108, and KF591109; *P. viburni*, KF591110, and KF591111; *P. ficus*, KF591106, and KF591107).

The ancient state of sites under concerted evolution was inferred for the last common ancestor (LCA) of “*Ca*. Tremblaya princeps” strains from clusters C and E. Multiple alignment was done with ClustalW (Larkin et al., 2007). Analysis was performed by Maximum Likelihood (ML) with the DNAML program of the PHYLIP v3.69 package (Felsenstein, 2005), predefining the tree topology as already determined (Hardy et al., 2008).

The 16S rRNA gene (locus *rrs*) sequences from 19 gammaproteobacterial endosymbionts of mealybugs were retrieved from GenBank and aligned with ClustalW (Larkin et al., 2007). Later edition with Gblocks 0.91b (Castresana, 2000) yielded a total of 1389 unambiguously aligned sites. Phylogenetic analyses were performed by ML, Maximum Parsimony (MP) and Bayesian inference (BI) using RAxML v8 (Stamatakis, 2014), DNAPARS from PHYLIP v3.69 package (Felsenstein, 2005), and MrBayes 3.2 (Ronquist et al., 2012), respectively. A separate general time-reversible evolutionary model with gamma-distributed rates and a proportion of invariant sites (GTR+I+G) was applied for ML and BI phylogenetic reconstructions, according to inferences by JModelTest 2 (Guindon and Gascuel, 2003; Darriba et al., 2012). ML and MP reconstructions included a 1000-replications bootstrap analysis. BI reconstruction was generated from two runs of 150,000 generations. Likelihood settings were set to nst = 6, rates = invgamma and ngammacat = 4. Sampling was performed every 100 generations. First 3400 generations were discarded as “burn in.” The phylogenetic analysis Figure was prepared using FigTree v1.4.0 (http://tree.bio.ed.ac.uk/software/figtree/) and Inkscape (https://inkscape.org/es/).

The complete genomes of “*Ca*. Tremblaya princeps” PCVAL and “*Ca*. Moranella endobia” PCVAL were scanned with plugin “Find Repeats” from Unipro UGENE v1.12.2 (Okonechnikov et al., 2012) to identify DNA repeats. Direct and inverted repeats (DR and IR, respectively) of at least 20 nucleotides in length were analyzed. GC content of independent repeats was calculated with MEGA5 (Tamura et al., 2011).

### Gene screening

Complete sequences of HR-related genes *recA* (encoding the recombination protein RecA), *recG* (encoding the ATP-dependent DNA helicase RecG, EC:3.6.4.12), *ruvA* (encoding the Holliday junction DNA helicase RuvA, EC:3.6.4.12), *ruvB* (encoding the Holliday junction DNA helicase RuvB, EC:3.6.4.12), *ruvC* (encoding the crossover junction endodeoxyribonuclease RuvC, EC:3.1.22.4), and *priA* (encoding the primosomal protein N', EC:3.6.4.-) were retrieved from GenBank for a set of selected betaproteobacteria (*Burkholderia glumae* BGR1, *B. multivorans* ATCC 17616, *B. pseudomallei* 1106a, and *B. thailandensis* E264) and gammaproteobacteria (*Escherichia coli* K-12 MG1655, *Salmonella enterica* Typhimurium LT2, *Serratia proteomaculans* 568, *Sodalis glossinidius*, *Dickeya dadantii* 3937, *Yersinia pestis* Angola, and “*Ca*. Moranella endobia” PCVAL). Multiple alignments were performed with ClustalW (Larkin et al., 2007) in order to identify conserved motifs where degenerate primers for PCR amplification and sequencing could be designed (Table S1). The annealing temperature in the PCR amplifications was 52°C. Most of the primer pairs (named as BG) are expected to amplify both beta- and gammaproteobacterial homologs of the targeted locus. Primers G-ruvAF/R were designed on a multiple alignment including gammaproteobacterial homologs only, in order to obtain a *ruvA* sequence larger than the one amplified by BG-ruvAF/R. BLAST searches against the non-redundant protein database (http://blast.ncbi.nlm.nih.gov/Blast.cgi/; Altschul et al., 1997) were performed in order to identify the putative taxonomic origin of the obtained sequences.

## Results and discussion

### The ancestral duplicated ribosomal genomic region in “*Ca*. tremblaya”

**Figure 1:**
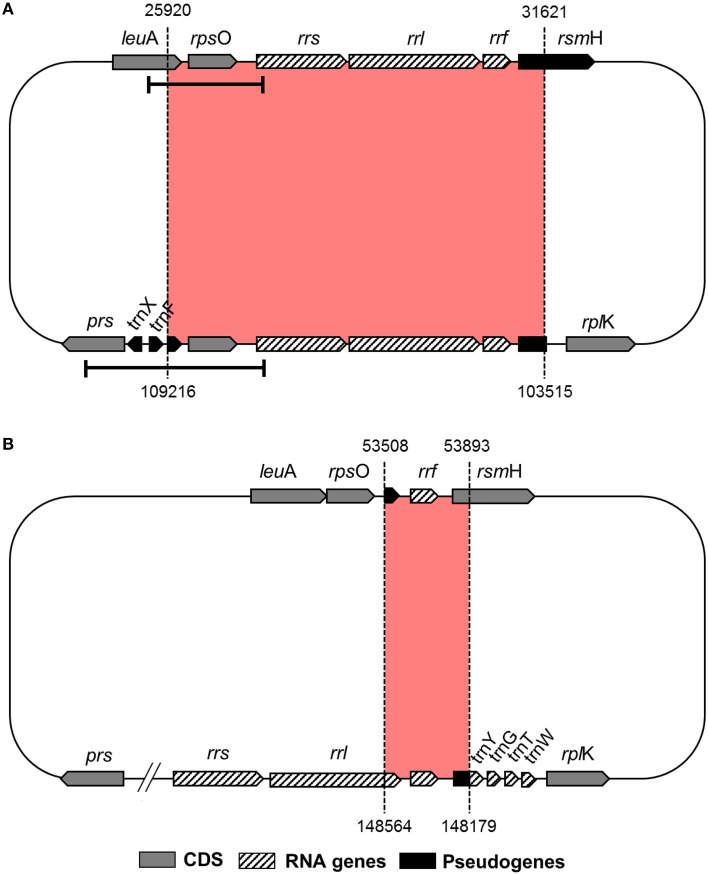
**Duplicated genomic regions in Pseudococcidae beta-endosymbionts**. **(A)** “*Ca*. Tremblaya princeps” PCVAL; **(B)** “*Ca*. Tremblaya phenacola” PAVE. Bars indicate the “*Ca*. Tremblaya princeps” fragments amplified in this work.

Reductive evolution in obligate endosymbiont genomes is mostly due to the loss of genes that become redundant and/or unnecessary in the intracellular niche (McCutcheon and Moran, 2012). However, even though “*Ca*. Tremblaya princeps” from *P. citri* (cluster E) displays one of the most reduced genomes known so far, it presents an identical 5702-bp redundant sequence. It includes the complete ribosomal operon (*rrs*, *rrl*, and *rrf*) and its closest genomic context (the 3′ region of *leu*A, encoding the alpha-isopropylmalate synthase, EC 2.3.3.13; *rps*O, encoding the ribosomal protein S15; and the 5′ region of *rsm*H, encoding the 16S rRNA m4C1402 methyltransferase, EC 2.1.1.199) (Figure 1). Detection of this duplicated region also in “*Ca*. Tremblaya princeps” strains from *Dysmicoccus brevipes* (cluster A), *Melanococcus albizziae* (cluster C), *Maconellicoccus australiensis*, and *Maconellicoccus hirsutus* (cluster F) led authors to suggest that a segmental duplication occurred at early stages of “*Ca*. Tremblaya princeps” diversification (Baumann et al., 2002). In order to study the origin of such duplication event, we performed a comparative analysis between the complete genomes of “*Ca*. Tremblaya princeps” PCVAL (López-Madrigal et al., 2011) and “*Ca*. Tremblaya phenacola” PAVE (Husnik et al., 2013). The analysis revealed the presence of an identical 386-bp inverted duplication in the latter. It is mostly composed by the remnants of the degraded ribosomal operon, including the 3′ end of a pseudogenized 23S rRNA gene (*rrl*, not annotated originally in the genome), the 5S rRNA gene (*rrf*) and the corresponding intergenic sequence. It also includes the TPPAVE_188 pseudogene, which is a truncated paralog of *rsmH* (Figure 1). This result suggests that the segmental duplication took place before the split of the two “*Ca*. Tremblaya” lineages. Moreover, the original copy of the ribosomal operon has undergone massive decay in “*Ca*. Tremblaya phenacola,” while the two identical copies preserved in “*Ca*. Tremblaya princeps” have evolved in a concerted manner.

### Co-occurrence of concerted evolution and HR-related genes in pseudococcinae endosymbiotic systems

Since both “*Ca*. Tremblaya” have a common evolutionary origin and “*Ca*. Tremblaya phenacola” has remained alone in the bacteriocytes of Phenacoccinae mealybugs (Gruwell et al., 2010; Koga et al., 2013), the massive decay of the paralogous loci in “*Ca*. Tremblaya phenacola” suggests that a link might exist between nested endosymbiosis and concerted evolution in “*Ca*. Tremblaya princeps.” The drastic reduction of the identical paralogous loci in “*Ca*. Tremblaya phenacola” PAVE co-occurs with additional genomic features that indicate a conventional reductive evolution (i.e., lower GC-content, high gene density; Husnik et al., 2013). No DNA repair and recombination genes were found in the genomes of “*Ca*. Tremblaya phenacola” PAVE or “*Ca*. Tremblaya princeps” from *P. citri* (López-Madrigal et al., 2011; McCutcheon and von Dohlen, 2011; Husnik et al., 2013), as it is typical for most endosymbionts with reduced genomes. In contrast, an almost complete set of HR-related loci were annotated in the genome of “*Ca*. Moranella endobia,” thus suggesting these genes to be responsible for the concerted evolution affecting “*Ca*. Tremblaya princeps” (López-Madrigal et al., 2013a). In order to test this hypothesis, we searched for the co-occurrence of signs of concerted evolution and the presence of HR-related genes in the endosymbiotic consortia from four unexplored mealybug species belonging to subfamily Pseudococcinae (*D. boninsis, P. longispinus, P. viburni*, and *P. ficus*).

#### Concerted evolution in “*Ca*. Tremblaya princeps”

**Figure 2:**
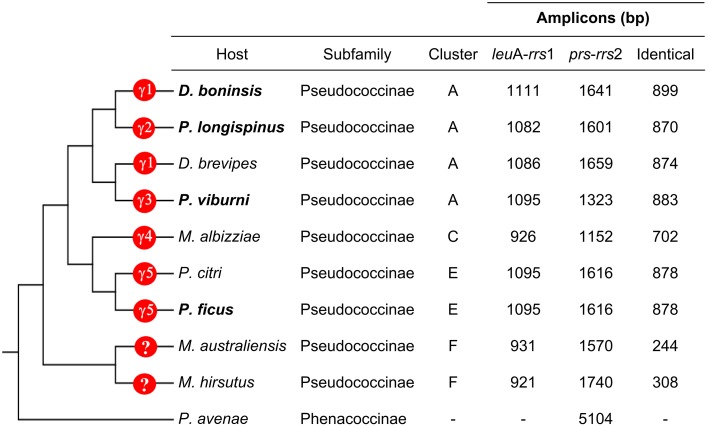
**Characteristics of the** ***leu*****A-*****rrs*****1 and** ***prs*****-*****rrs*****2 regions**. Host species from which sequences have been obtained in this work are in bold. The phylogenetic relationship among the insect hosts (Hardy et al., 2008), as well as the presence of gammaproteobacteria in the corresponding endosymbiotic systems are indicated. γ1 to γ5 represent polyphyletic bacterial lineages (see Section Genetic Screening of HR-related Genes).

To search for signals of concerted evolution, we focused on the molecular analysis of the 5′-flanking regions of the duplicated ribosomal operons (*leuA-rrs1* and *prs-rrs2*) in the four “*Ca*. Tremblaya princeps” strains under study. The obtained amplicons include the 3′-end of *leuA*, an almost complete *prs* (encoding the phosphoribosylpyrophosphate synthetase, EC 2.7.6.1), the complete sequence of *rpsO*, several tRNA genes and the 5′-end of *rrs* (Figures 1, 2). The alignment of the amplified sequences revealed the existence of identical paralogous fragments ranging from 870 bp in “*Ca*. Tremblaya princeps” strain PLON (beta-endosymbiont of *P. longispinus*) to 899 bp in strain DBON (beta-endosymbiont of *D. boninsis*). Comparative analyses with available orthologous sequences of “*Ca*. Tremblaya princeps” strains from *D. brevipes*, *P. citri*, and *M. albizziae* (Baumann et al., 2002) showed that the length of these regions under concerted evolution remains relatively homogeneous (702–899 bp) among “*Ca*. Tremblaya princeps” lineages from clusters A, E, and C. In agreement with their close evolutionary relationship, identical duplicated loci start at orthologous positions for all available members of cluster A (nucleotide 25915/109218 in “*Ca*. Tremblaya princeps” PCVAL) and cluster E (nucleotides 25920/109216 in strain PCVAL; Figure 1), respectively. In contrast, identical loci are drastically reduced in strains from *M. australiensis* and *M. hirsutus* (cluster F; Baumann et al., 2002), whose initial nucleotides are orthologous of sites 26557/108579 and 26387/108749 in strain PCVAL, respectively. As above indicated, no nested intracellular bacteria have been reported in cluster F (Thao et al., 2002). However, no microscopic exploration of endosymbiotic systems from cluster F has been performed and, therefore, the presence of an undetected gamma-endosymbionts cannot be ruled out.

*P. citri* and *P. ficus* are so closely related that they have been considered as cryptic species (Kol-Maimon et al., 2014). The comparison of the identical paralogous regions in the genomes of their “*Ca*. Tremblaya princeps” strains revealed homogenization of polymorphisms within each strain. Four indels and (at least) 15 nucleotide substitutions were detected (Table 1). In order to characterize the mutations leading to these homogenized polymorphic sites, their ancestral state in the LCA of “*Ca*. Tremblaya princeps” of clusters C and E was inferred with over 95% probability (Table 1). These data suggest ongoing homogenization by concerted evolution between the duplicated copies, at least in “*Ca*. Tremblaya princeps” from cluster E.

**Table 1:** **Homogenized polymorphisms in “*****Ca.*** **Tremblaya princeps” strains from** ***P. ficus*** **(PFIC) and** ***P. citri*** **(PCVAL)**.

**Cluster A**				**Cluster C**	**Cluster E**		**LCA (C, E)**	**Genome position**	
**DBON**	**PLON**	**DBRE**	**PVIB**	**MALB**	**PFIC**	**PCVAL**		***leuA*-rrs1**	***prs*-rrs2**
G	–	G	G	G	C	A	G	25,955	109,181
C	–	C	C	C	C	G	C	25,957	109,179
T	–	T	T	T	T	G	T	25,961	109,175
C	–	C	C	C	C	A	C	25,962	109,176
T	T	A	T	T	T	G	T	26,057	109,079
T	T	T	T	T	C	G	T	26,102	109,034
G	G	G	G	G	C	A	G	26,106	109,030
G	G	G	G	G	T	A	G	26,107	109,029
A	A	–	A	–	A	T	A	26,417	108,719
T	T	T	G	–	T	A	T	26,437	108,699
G	G	G	C	–	G	C	G	26,438	108,698
T	T	T	A	–	T	A	T	26,439	108,697
C	C	C	C	–	–	C	C	26,479	108,657
A	A	A	A	–	A	–	A	26,479–80	108,656–7
G	G	G	G	–	G	–	G	26,479–80	108,656–7
C	C	C	C	–	–	C	C	26,480	108,656
C	C	C	C	–	A	C	C	26,482	108,654
T	T	T	T	–	T	G	T	26,494	108,642
T	T	T	T	T	G	T	T	26,526	108,610

*The genome position indicates the corresponding nucleotide along the “Ca. Tremblaya princeps” PCVAL genome. The state of orthologous sites in the analyzed strains from clusters A, C, and E, as well as the most likely nucleotide in the LCA of clusters C and E, is represented. Nucleotide substitutions from the LCA are in red. DBON, D. boninsis; PLON, P. longispinus; DBRE, D. brevipes; PVIB, P. viburni; MALB, M. albizziae*.

#### Genetic screening of HR-related genes

In order to explore the HR potential of the endosymbiotic consortia from the four analyzed Pseudococcinae species, we investigated the presence of a set of HR-related genes already identified in the genome of “*Ca*. Moranella endobia” from *P. citri*. Screened loci include *recA*, *recG*, *ruvA*, *ruvB*, *ruvC*, and *priA*. Most of them (*recA*, *recG, ruvA*, *ruvB*, *ruvC*) are common elements of both RecF and RecBCD pathways (Rocha et al., 2005; Spies and Kowalczykowski, 2005). RecG may functionally replace RuvABC (Meddows et al., 2004). In contrast, PriA is exclusively involved in the RecBCD pathway (Ng and Marians, 1996) and has been proposed to catalyze the assembly of the “*Ca*. Moranella endobia” incomplete primosome (López-Madrigal et al., 2013a).

The results are presented in Table 2. The GenBank accession numbers for all newly amplified sequences are also indicated. BLAST searches against the non-redundant protein database suggest a gammaproteobacterial origin for the loci detected in *P. ficus*, *P. longispinus*, and *P. viburni*. They show best similarity hits with homologs from bacteria of genus “*Ca*. Moranella”, *Sodalis* and *Pectobacterium*, respectively. Identical best similarity hits were observed when using their16S rRNA genes (AF476108, KF742539, JN182341) as query sequences. These results indicate that the internalization of the corresponding gamma-endosymbiont made recurrently available an HR machinery to the long-term endosymbiont “*Ca*. Tremblaya princeps.”

**Table 2:** **Genetic screening of selected homologous recombination genes**.

	***P. citri***	***P. ficus***	***P. longispinus***	***P. viburni***	***D. boninsis***
*recA*	+	+ (KJ140122)	−	−	−
*recG*	+	+ (KJ140123)	−	−	−
*ruvA*	+	+ (KJ140120)	+ (KJ140121)	−	−
*ruvB*	+	+ (KJ140117)	+ (KJ140118)	+ (KJ140119)	−
*ruvC*	+	+ (KJ140124)	−	−	−
*priA*	+	−	+ (KJ140115)	+ (KJ140116)	−

*+, detected gene; −, non-detected gene*.

Although all primer combinations successfully amplified their target when applied to *P. citri* as a positive control, none of the analyzed consortia gave positive results for all screened genes. Negative results should be interpreted with caution, since they do not necessarily imply the absence of undetected loci. Degenerate primers were designed on gene regions encoding highly conserved motifs among beta and gammaproteobacterial homologs of the analyzed genes (Table S1). However, although highly conserved between distantly related bacteria, motifs acting as primer templates are not directly involved in protein functionality. Therefore, it is possible that non-synonymous substitutions affecting the target sequence lead to false negative results. Nevertheless, in accordance with the close evolutionary relationship between *P. citri* and *P. ficus*, five of the six screened loci were detected in the latter. Only *priA* could not be detected. PriA is needed for the assembly of the primosome, which is already incomplete in “*Ca*. Moranella endobia” PCVAL, due to the loss of *dnaT* and *priC* (López-Madrigal et al., 2013a). Thus, its absence suggests a relatively recent inactivation of the RecBCD pathway in the nested endosymbiont of “*Ca*. Tremblaya princeps” strains from cluster E. In contrast, as revealed by the very recent homogenization of polymorphisms (Table 1), the RecF pathway appears to be still acting on this cluster. Nevertheless, RecF function is expected to be attenuated because none of the components of the RecFOR complex, which enhances RecA loading onto SSB-coated single stranded DNA (Morimatsu and Kowalczykowski, 2003; Handa et al., 2009), is present in “*Ca*. Moranella endobia” PCVAL. Furthermore, *rec*A mutations known to bypass the RecFOR complex deficiency (i.e., recA441, recA730, recA803; Lavery and Kowalczykowski, 1992) were not detected in that genome.

**Figure 3:**
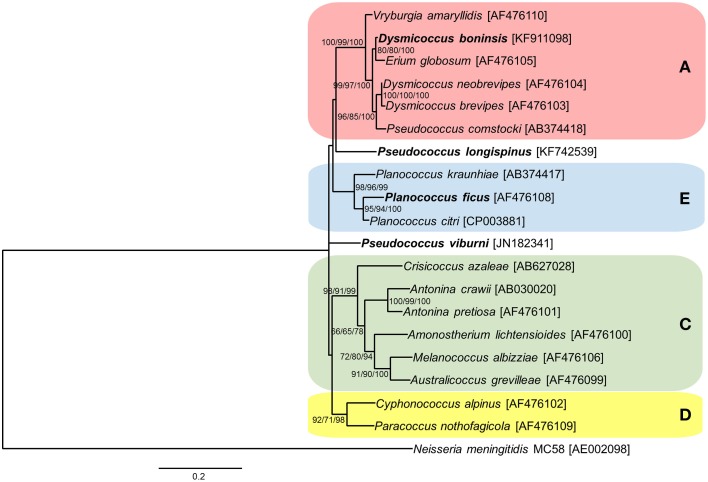
**Phylogenetic relationships among gamma-endosymbionts of Pseudococcinae mealybugs**. Already defined clusters **A** (red), **C** (green), **D** (yellow), and **E** (blue) are represented. Species used in this work are in bold. The betaproteobacterium *Neisseria meningitidis* MC58 was used as outgroup. ML, MP, and BI analysis gave essentially the same results. ML and MP bootstrap values, and Bayesian posterior probabilities over 50% are indicated. Scale bar represents substitutions per site.

As for the endosymbiotic consortia involving the three “*Ca*. Tremblaya princeps” strains from cluster A under study (*D. boninsis*, *P. longispinus*, and *P. viburni*), our results suggest that both RecF and RecBCD pathways are currently inactive. Different patterns of conservation of HR-related genes were observed, which is consistent with the independent evolutionary origin of the gamma-endosymbionts (Gatehouse et al., 2012; López-Madrigal et al., 2014). Cluster A represents a very wide clade, including betaproteobacterial endosymbionts from mealybugs of the tribe Pseudococcini and the southern Africa group (Thao et al., 2002; Hardy et al., 2008). Moreover, *Pseudococcus* is a polyphyletic genus, and the two species analyzed in this work are phylogenetically distant, belonging to different clades of the tribe Pseudococcini. In order to place the three gamma-endosymbionts of these insects in the phylogenetic tree of those already described for mealybugs, we performed a phylogenetic analysis based on 16S rDNA sequences (Figure 3). According to our results, only the gamma-endosymbiont of *D. boninsis* groups with the other nested endosymbionts of “*Ca*. Tremblaya princeps” strains from cluster A, showing a long co-evolutionary history with its symbiotic partner. In contrast, the gamma-endosymbionts of *P. longispinus* and *P. viburni* group neither with any other cluster nor between them. The present analysis suggests the replacement of the ancestral gamma-endosymbiont in these two *Pseudococcus* species, and reveals two independent events of HR-related genes acquisition by the corresponding “*Ca*. Tremblaya princeps” strains. As expected for recently acquired obligate symbionts, these gamma-endosymbionts appear to be less affected by reductive evolution than that of *D. boninsis*, where none of the screened genes had been detected. Nevertheless, even if HR pathways appear to be currently inactive in the analyzed members of cluster A, this is not inconsistent with the observed signs of concerted evolution in the corresponding “*Ca*. Tremblaya princeps” strains (Figure 2). Signs of concerted evolution do not necessarily co-exist with functional HR pathways, since repeated identical sequences are expected to last on the genome over a certain time after the inactivation of such pathways. The presence of identical paralogous loci has also been noticed in the genome of “*Ca*. Portiera aleyrodidarum,” obligate endosymbiont of the whitefly *Bemisia tabaci*, where HR pathways have been recently lost (Sloan and Moran, 2013).

### Susceptibility to homologous recombination of nested endosymbionts from *P. citri*

Due to the reductive genome evolution in obligatory endosymbionts, genetic essentiality in their functional networks is typically higher than that observed in free-living bacteria (Thomas et al., 2009). Therefore, HR events and associated genome deletions or rearrangements could dramatically risk the stability of bacterial consortia involving tiny genomes. Repeat sequences ranging from 18 to 24 bp are thought to be long enough to promote HR events (Shen and Huang, 1986; Aras et al., 2003; Sloan and Moran, 2013). Therefore, in order to analyze the susceptibility to HR of both “*Ca*. Tremblaya princeps” and “*Ca*. Moranella endobia” from *P. citri* we performed a comprehensive search for direct (DR) and inverted (IR) repeats with at least 20 bp in length in both genomes (Table S2). Sixteen DRs (TDR01 to 16) and 12 IRs (TIR01 to 12) were found in “*Ca*. Tremblaya princeps.” Except for TIR12 (i.e., the duplicated region containing the ribosomal operon), all other repeats seem to have been randomly generated. As for “*Ca*. Moranella endobia”, 24 DRs (MDR01 to 24) and 16 IRs (MIR01 to 16) were found. Several of them appear to be consequence of ancestral duplications. Thus, MDR01, MDR02, and MDR11 map on a functional *pdx*J (locus MPC_094 in the genome) and its pseudogenized copy (MPC_306), while MDR07, MDR14-16, MDR19, MDR22, and MDR23 are linked to a duplication including genes *sec*E (MPC_278) and *tuf* (MPC_279). Additionally, seven DRs and five IRs map on several tRNA loci, which mostly display highly similar anticodon sequences and whose relative orientation along the genome is consistent with an ancestral proliferation process (Withers et al., 2006). Conservation of these repeats might be linked to mutational constraints, since 36–71% of their sequences correspond to tRNA stem regions (Table S3).

**Figure 4:**
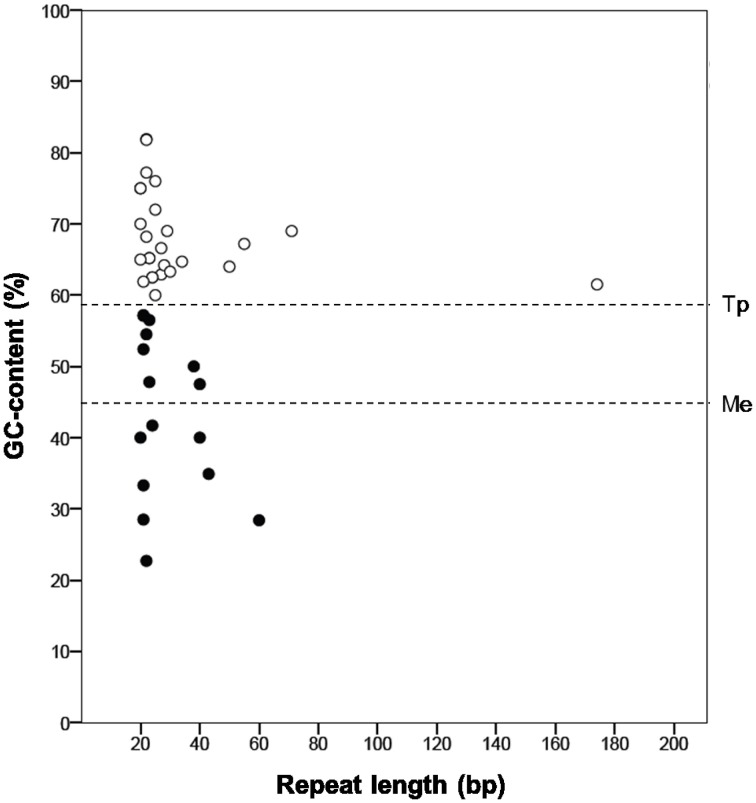
**Molecular characterization of independent repeats**. Those identified in the genomes of “*Ca*. Tremblaya princeps” (white circles) and “*Ca*. Moranella endobia” (black circles) are represented. The horizontal lines indicate the mean GC-content of each genome.

“*Ca*. Tremblaya princeps” repeats abundance is likely linked to its high genomic GC-content (Figure 4). The molecular characterization of independently generated repeats identified in these genomes reveals that those of “*Ca*. Tremblaya princeps” are GC-enriched compared to the whole genome (GC_repeats_ = 67.7% versus GC_genome_ = 59%, SD_G+C_ = 6.5), while no bias is observed in the case of “*Ca*. Moranella endobia” (GC_repeats_ = 42.4% versus GC_genome_ = 44%, SD_G+C_ = 11.0).

According to our results, sequence repeats are larger in “*Ca*. Tremblaya princeps” (mean length = 235.9 bp) than in *Ca*. Moranella endobia” (mean length = 127.6 bp). In addition, some of them (TDR8 and TDR12; TIR05 and TIR07) appear to derive from larger ancestral repeats. They are also more abundant in the former, where repeats density (abundance/kb) is 2.85 times larger than that of “*Ca*. Moranella endobia” (Table S2). Therefore, “*Ca*. Tremblaya princeps” must be more sensitive to HR than “*Ca*. Moranella endobia” (Rocha, 2003). In spite of this, HR events are not expected to be highly frequent in “*Ca*. Tremblaya princeps.” Recombination between DRs would cause DNA deletions or DNA duplications. Taking into account that the mean distance between DRs is about 50 kb (36% of the chromosome), further genome reduction would be strongly deleterious. On the other hand, recombination mediated by IRs would generate DNA inversions. Half of the IRs detected in the “*Ca*. Tremblaya princeps” genome map on relevant loci, including genes involved in translation (*rplS*, *rpsF*, *rpmA*) and essential amino acids biosynthesis (*pheA*, *ilvI*, *aroB*), whose functionality might be seriously compromised by HR events (Table S2). Thus, the apparent inactivation of the HR pathways in *D. boninsis*, *P. longispinus*, and *P. viburni* or its attenuation in *P. ficus* and *P. citri* may be helping to maintain the stability of the corresponding endosymbiotic systems.

In summary, our work reveals that the segmental duplication involving the ribosomal operon took place before the divergence between “*Ca*. Tremblaya princeps” and “*Ca*. Tremblaya phenacola.” Strikingly, there is a drastic reduction of the identical paralogous loci in the genome of “*Ca*. Tremblaya phenacola” PAVE. This is consistent with the apparently conventional reductive evolution undergone by this bacterium and suggest a link between concerted evolution and nested endosymbiosis. Results from the genetic screening indicate that independent internalization of different gamma-endosymbionts allowed the recurrent acquisition of HR capabilities by the corresponding endosymbiotic systems. Nevertheless, HR pathways appear to be currently attenuated or inactivated in the tested mealybug species, which could be enhancing the stability of these bacterial consortia. A metagenomic-based approach leading to the complete genomic characterization of the analyzed bacterial consortia would be useful in order to confirm our results.

### Conflict of interest statement

The authors declare that the research was conducted in the absence of any commercial or financial relationships that could be construed as a potential conflict of interest.

## References

[B1] Abby S. Daubin V. (2007). Comparative genomics and the evolution of prokaryotes. Trends Microbiol. 15, 135–141. 10.1016/j.tim.2007.01.007 17289390

[B2] Altschul S. F. Madden T. L. Schäffer A. A. Zhang J. Zhang Z. Miller W.. (1997). Gapped BLAST and PSI-BLAST: a new generation of protein database search programs. Nucleic Acids Res. 25, 3389–3402. 10.1093/nar/25.17.3389 9254694 PMC146917

[B3] Aras R. A. Kang J. Tschumi A. I. Harasaki Y. Blaser M. J. (2003). Extensive repetitive DNA facilitates prokaryotic genome plasticity. Proc. Natl. Acad. Sci. U.S.A. 100, 13579–13584. 10.1073/pnas.1735481100 14593200 PMC263856

[B4] Baumann L. Thao M. L. Hess J. M. Johnson M. W. Baumann P. (2002). The genetic properties of the primary endosymbionts of mealybugs differ from those of other endosymbionts of plant sap-sucking insects. Appl. Environ. Microbiol. 68, 3198–3205. 10.1128/AEM.68.7.3198 12088995 PMC126778

[B5] Casjens S. (1998). The diverse and dynamic structure of bacterial genomes. Annu. Rev. Genet. 32, 339–377. 10.1146/annurev.genet.32.1.339 9928484

[B6] Castresana J. (2000). Selection of conserved blocks from multiple alignments for their use in phylogenetic analysis. Mol. Biol. Evol. 17, 540–552. 10.1093/oxfordjournals.molbev.a026334 10742046

[B7] Darriba D. Taboada G. L. Doallo R. Posada D. (2012). jModelTest 2: more models, new heuristics and parallel computing. Nat. Methods 9, 772. 10.1038/nmeth.2109 22847109 PMC4594756

[B8] Felsenstein J. (2005). Using the quantitative genetic threshold model for inferences between and within species. Philos. Trans. R. Soc. Lond. B Biol. Sci. 360, 1427–1434. 10.1098/rstb.2005.1669 16048785 PMC1569509

[B9] Gatehouse L. N. Sutherland P. Forgie S. A. Kaji R. Christeller J. T. (2012). Molecular and histological characterization of primary (betaproteobacteria) and secondary (gammaproteobacteria) endosymbionts of three mealybug species. Appl. Environ. Microbiol. 78, 1187–1197. 10.1128/AEM.06340-11 22156418 PMC3273002

[B10] Gruwell M. E. Hardy N. B. Gullan P. J. Dittmar K. (2010). Evolutionary relationships among primary endosymbionts of the mealybug subfamily phenacoccinae (Hemiptera: Coccoidea: Pseudococcidae). Appl. Environ. Microbiol. 76, 7521–7525. 10.1128/AEM.01354-10 20851962 PMC2976180

[B11] Guindon S. Gascuel O. (2003). A simple, fast, and accurate algorithm to estimate large phylogenies by maximum likelihood. Syst. Biol. 52, 696–704. 10.1080/10635150390235520 14530136

[B12] Handa N. Morimatsu K. Lovett S. T. Kowalczykowski S. C. (2009). Reconstitution of initial steps of dsDNA break repair by the RecF pathway of *E. coli. Genes Dev*. 23, 1234–1245. 10.1101/gad.1780709 19451222 PMC2685532

[B13] Hardy N. B. Gullan P. J. Hodgson C. J. (2008). A subfamily-level classification of mealybugs (Hemiptera: Pseudococcidae) based on integrated molecular and morphological data. Syst. Entomol. 33, 51–71. 10.1111/j.1365-3113.2007.00408.x

[B14] Husnik F. Nikoh N. Koga R. Ross L. Duncan R. P. Fujie M.. (2013). Horizontal gene transfer from diverse bacteria to an insect genome enables a tripartite nested mealybug symbiosis. Cell 153, 1567–1578. 10.1016/j.cell.2013.05.040 23791183

[B15] Koga R. Nikoh N. Matsuura Y. Meng X. Y. Fukatsu T. (2013). Mealybugs with distinct endosymbiotic systems living on the same host plant. FEMS Microbiol. Ecol. 83, 93–100. 10.1111/j.1574-6941.2012.01450.x 22809388

[B16] Kol-Maimon H. Ghanim M. Franco J. C. Mendel Z. (2014). Evidence for gene flow between two sympatric mealybug species (Insecta; Coccoidea; Pseudococcidae). PLoS ONE 9:e88433. 10.1371/journal.pone.0088433 24523894 PMC3921159

[B17] Kono M. Koga R. Shimada M. Fukatsu T. (2008). Infection dynamics of coexisting beta- and gammaproteobacteria in the nested endosymbiotic system of mealybugs. Appl. Environ. Microbiol. 74, 4175–4184. 10.1128/AEM.00250-08 18469124 PMC2446506

[B18] Larkin M. A. Blackshields G. Brown N. P. Chenna R. McGettigan P. A. McWilliam H.. (2007). Clustal W and Clustal X version 2.0. Bioinformatics 23, 2947–2948. 10.1093/bioinformatics/btm404 17846036

[B19] Laslett D. Canback B. (2004). ARAGORN, a program to detect tRNA genes and tmRNA genes in nucleotide sequences. Nucleic Acids Res. 32, 11–16. 10.1093/nar/gkh152 14704338 PMC373265

[B20] Lavery P. E. Kowalczykowski S. C. (1992). Biochemical basis of the constitutive repressor cleavage activity of recA730 protein. A comparison to recA441 and recA803 proteins. J. Biol. Chem. 267, 20648–20658. 1400384

[B21] Liao D. (1999). Concerted evolution: molecular mechanism and biological implications. Am. J. Hum. Genet. 64, 24–30. 10.1086/302221 9915939 PMC1377698

[B22] López-Madrigal S. Balmand S. Latorre A. Heddi A. Moya A. Gil R. (2013a). How does *Tremblaya princeps* get essential proteins from its nested partner *Moranella endobia* in the mealybug *Planoccocus citri*? PLoS ONE 8:e77307. 10.1371/journal.pone.0077307 24204799 PMC3804617

[B23] López-Madrigal S. Beltrà A. Resurrección S. Soto A. Latorre A. Moya A.. (2014). Molecular evidence for ongoing complementarity and horizontal gene transfer in endosymbiotic systems of mealybugs. Front. Microbiol. 5:449. 10.3389/fmicb.2014.00449 25206351 PMC4144094

[B24] López-Madrigal S. Latorre A. Porcar M. Moya A. Gil R. (2011). Complete genome sequence of “*Candidatus* Tremblaya princeps” strain PCVAL, an intriguing translational machine below the living-cell status. J. Bacteriol. 193, 5587–5588. 10.1128/JB.05749-11 21914892 PMC3187454

[B25] López-Madrigal S. Latorre A. Porcar M. Moya A. Gil R. (2013b). Mealybugs nested endosymbiosis: going into the “matryoshka” system in *Planococcus citri* in depth. BMC Microbiol. 13:74. 10.1186/1471-2180-13-74 23548081 PMC3620526

[B26] Lowe T. M. Eddy S. R. (1997). tRNAscan-SE: a program for improved detection of transfer RNA genes in genomic sequence. Nucleic Acids Res. 25, 955–964. 10.1093/nar/25.5.0955 9023104 PMC146525

[B27] McCutcheon J. P. Moran N. A. (2012). Extreme genome reduction in symbiotic bacteria. Nat. Rev. Microbiol. 10, 13–26. 10.1038/nrmicro2670 22064560

[B28] McCutcheon J. P. von Dohlen C. D. (2011). An interdependent metabolic patchwork in the nested symbiosis of mealybugs. Curr. Biol. 21, 1366–1372. 10.1016/j.cub.2011.06.051 21835622 PMC3169327

[B29] Meddows T. R. Savory A. P. Lloyd R. G. (2004). RecG helicase promotes DNA double-strand break repair. Mol. Microbiol. 52, 119–132. 10.1111/j.1365-2958.2003.03970.x 15049815

[B30] Morimatsu K. Kowalczykowski S. C. (2003). RecFOR proteins load RecA protein onto gapped DNA to accelerate DNA strand exchange: a universal step of recombinational repair. Mol. Cell. 11, 1337–1347. 10.1016/S1097-2765(03)00188-6 12769856

[B31] Moya A. Gil R. Latorre A. (2009). The evolutionary history of symbiotic associations among bacteria and their animal hosts: a model. Clin. Microbiol. Infect. 15(Suppl. 1), 11–13. 10.1111/j.1469-0691.2008.02689.x 19220345

[B32] Moya A. Peretó J. Gil R. Latorre A. (2008). Learning how to live together: genomic insights into prokaryote-animal symbioses. Nat. Rev. Genet. 9, 218–229. 10.1038/nrg2319 18268509

[B33] Ng J. Y. Marians K. J. (1996). The ordered assembly of the FX174-type primosome I. Isolation and identification of intermediate protein-DNA complexes. J. Biol. Chem. 271, 15642–15648. 10.1074/jbc.271.26.15642 8663104

[B34] Okonechnikov K. Golosova O. Fursov M. (2012). Unipro UGENE: a unified bioinformatics toolkit. Bioinformatics 28, 1166–1167. 10.1093/bioinformatics/bts091 22368248

[B35] Rocha E. P. C. (2003). DNA repeats lead to the accelerated loss of gene order in bacteria. Trends Genet. 19, 600–603. 10.1016/j.tig.2003.09.011 14585609

[B36] Rocha E. P. C. (2008). The organization of the bacterial genome. Annu. Rev. Genet. 42, 211–233. 10.1146/annurev.genet.42.110807.091653 18605898

[B37] Rocha E. P. C. Cornet E. Michel B. (2005). Comparative and evolutionary analysis of the bacterial homologous recombination systems. PLoS Genet. 1:e15. 10.1371/journal.pgen.0010015 16132081 PMC1193525

[B38] Ronquist F. Teslenko M. van der Mark P. Ayres D. L. Darling A. Höhna S.. (2012). MrBayes 3.2: efficient Bayesian phylogenetic inference and model choice across a large model space. Syst. Biol. 61, 539–542. 10.1093/sysbio/sys029 22357727 PMC3329765

[B39] Rutherford K. Parkhill J. Crook J. Horsnell T. Rice P. Rajandream M. A.. (2000). Artemis: sequence visualization and annotation. Bioinformatics 16, 944–945. 10.1093/bioinformatics/16.10.944 11120685

[B40] Shen P. Huang H. V. (1986). Homologous recombination in *Escherichia coli*: dependence on substrate length and homology. Genetics 112, 441–457. 3007275 10.1093/genetics/112.3.441 PMC1202756

[B41] Sloan D. B. Moran N. A. (2013). The evolution of genomic instability in the obligate endosymbionts of whiteflies. Genome Biol. Evol. 5, 783–793. 10.1093/gbe/evt044 23542079 PMC3673631

[B42] Spies M. Kowalczykowski S. C. (2005). Homologous recombination by the RecBCD and the RecF pathways, in The Bacterial Chromosome, ed Higgins N. P. (Washington, DC: ASM Press), 389–403.

[B43] Staden R. Beal K. F. Bonfield J. K. (2000). The Staden package, 1998. Methods Mol. Biol. 132, 115–130. 10.1385/1-59259-192-2:115 10547834

[B44] Stamatakis A. (2014). RAxML version 8: a tool for phylogenetic analysis and post-analysis of large phylogenies. Bioinformatics 30, 1312–1313. 10.1093/bioinformatics/btu033 24451623 PMC3998144

[B45] Tamura K. Peterson D. Peterson N. Stecher G. Nei M. Kumar S. (2011). MEGA5: molecular evolutionary genetics analysis using maximum likelihood, evolutionary distance, and maximum parsimony methods. Mol. Biol. Evol. 28, 2731–2739. 10.1093/molbev/msr121 21546353 PMC3203626

[B46] Thao M. Gullan P. Baumann P. (2002). Secondary (γ-Proteobacteria) endosymbionts infect the primary (β-Proteobacteria) endosymbionts of mealybugs multiple times and coevolve with their hosts. Appl. Environ. Microbiol. 68, 3190–3197. 10.1128/AEM.68.7.3190 12088994 PMC126777

[B47] Thomas G. H. Zucker J. Macdonald S. J. Sorokin A. Goryanin I. Douglas A. E. (2009). A fragile metabolic network adapted for cooperation in the symbiotic bacterium *Buchnera aphidicola*. BMC Syst. Biol. 3:24. 10.1186/1752-0509-3-24 19232131 PMC2649895

[B48] von Dohlen C. D. Kohler S. Alsop S. T. McManus W. R. (2001). Mealybug β-proteobacterial endosymbionts contain γ-proteobacterial symbionts. Nature 412, 433–436. 10.1038/35086563 11473316

[B49] Withers M. Wernisch L. dos Reis M. (2006). Archaeology and evolution of transfer RNA genes in the *Escherichia coli* genome. RNA 12, 933–942. 10.1261/rna.2272306 16618964 PMC1464854

