# Supplementary material for: The link between independent acquisition of intracellular gamma-endosymbionts and concerted evolution in Tremblaya princeps
Source: Front Microbiol. 2015 Jun 25;6:642. doi: 10.3389/fmicb.2015.00642 (PMC4479817; doi:10.3389/fmicb.2015.00642)
Supplement: Supplementary file 1 [file DataSheet1.PDF]

Research article
June 8, 2015

Supplementary Material

The link between independent acquisition of intracellular gamma-endosymbionts and concerted evolution in *Tremblaya princeps*

Sergio López-Madrigal[1], Amparo Latorre[1,2], Andrés Moya[1,2], Rosario Gil[1]*

[1]Institut Cavanilles de Biodiversitat i Biologia Evolutiva (ICBiBE), Universitat de València, Paterna (València), Spain.
[2]Área de Genómica y Salud de la Fundación para el Fomento de la Investigación Sanitaria y Biomédica de la Comunitat Valenciana (FISABIO) – Salud Pública, València, Spain.

*** Correspondence:** Dr. Rosario Gil, Institut Cavanilles de Biodiversitat i Biologia Evolutiva, Universitat de València, C/ Catedrático José Beltrán 2, 46980 Paterna (Valencia), Spain.
rosario.gil@uv.es

Supplementary Tables

Table S1. Degenerate primers designed for the gene screening. Primers degeneration was calculated as the proportion of ambiguous sites. The conserved motifs position refers to the *Escherichia coli* homologous proteins.

Gene	Primer	Sequence (5´→3´)	Length (nt)	Degeneration	Conserved motif	Position
recA	BG-recAF	TTYGGNAARGGNWSNATHATG	21	38%	FGKGSIM	22-28
	BG-recAR	RTANSWRTACCANGCNCCNGC	21	38%	AGAWYSY	288-294
recG	BG-recGF	MWNGAYYTNBTNYTNCAYYTNCC	23	56%	Q/IDLL/VLHLP	34-41
	BG-recGR	GGNCCNCKDATYTCNARRTC	20	40%	DLEIRGP	626-632
ruvA	BG-ruvAF	GARGAYGCNCANYTNYTNTWYGG	23	43%	EDAQ/HLLY/FG	55-62
	BG-ruvAR	ARNCKYTCNGCNGTYTTYTTNCC	23	39%	GKKTAERL	117-124
	G-ruvAF	ACNTGYTTYTAYGMNYTNCC	20	40%	TCFYE/ALP	33-39
	G-ruvAR	ACNADNCKNSHNGCYTCYTG	20	50%	QEAS/GRM/LV	175-181
ruvB	BG-ruvBF	CCNCCNGGNYTNGGNAARACNAC	23	34%	PPGLGKTT	63-70
	BG-ruvBR	KNGGNGTNCKYTGNADRAANCC	22	45%	GFL/IQRTPR	309-316
ruvC	BG-ruvCF	GAYCCNGGNHBNMGNRTNACNGG	23	47%	DPGS/LRV/ITG	8-15
	BG-ruvCR	TGNSWDATNGCNRTNSCNARNGC	23	52%	ALA/GI/T/MAIT/CH	143-150
priA	BG-priAF	GGNRTNACNGGNWSNGGNAARAC	23	43%	GV/ITGSGKT	224-231
	BG-priAR	CCYTTNGCNADCATYTGNGTNCC	23	30%	GTQML/IAKG	538-545

Table S2. Repetitive sequences detected in the genomes of the *P. citri* endosymbionts. Sequences have been sorted by size. Information about their position and distribution (distance), as well as if they map on coding (gene name) or non-coding (-) regions, is provided.

Organism	Type	ID	Position 1	Position 2	Size (bp)	Distance (bp)	Context
"Ca. Tremblaya princeps*"*	DR	TDR01	776-795	55251-55270	20	54455	-/*lysC*
		TDR02	3058-3077	53837-53856	20	50759	-/-
		TDR03	16248-16267	97630-97649	20	81362	-/*rpoC*
		TDR04	44279-44300	79739-79760	22	35438	-/*rpo*A
		TDR05	58673-58694	59973-59994	22	1278	-/-
		TDR06	6373-6395	21488-21510	23	15092	-/*dnaE*
		TDR07	63473-63495	126023-126045	23	62527	-/*clpX*
		TDR08	58059-58082	121811-121834	24	63728	*trnK*1/-
		TDR09	101936-101959	121556-121579	24	19596	*rpoB*/-
		TDR10	69981-70005	111177-111201	25	41171	*engA*/-
		TDR11	15006-15032	121818-121844	27	106785	*trnW*/*rpoD*
		TDR12	58103-58129	121833-121859	27	63703	*trnK*1/-
		TDR13	51226-51253	56851-56878	28	5597	*aroC*/-
		TDR14	73171-73220	130598-130647	50	57377	*trnK*2/-
		TDR15	47369-47423	69420-69474	55	21996	*infB*/*engA*
		TDR16	8141-8314	128322-128495	174	120007	-/*argG*
	IR	TIR01	22754-22773	43644-43663	20	20870	*ilvI*/*rpsF*
		TIR02	45099-45119	52235-52255	21	7115	-/-
		TIR03	52182-52202	110655-110675	21	58452	-/-
		TIR04	13832-13853	14058-14079	22	204	*rplS*/*rplS*
		TIR05	18479-18500	60398-60419	22	41897	-/-
		TIR06	10513-10537	66135-66159	25	55597	*pheA*/-
		TIR07	18448-18472	60427-60451	25	41954	-/-
		TIR08	78375-78403	121525-121553	29	43121	*aroB*/-
		TIR09	34369-34398	50394-50423	30	15995	-/-
		TIR10	32831-32864	60265-60298	34	27400	*rpmA*/-
		TIR11	56533-56603	63636-63706	71	7032	-/-
		TIR12	25920-31621	103515-109216	5702	71893	*
"Ca. Moranella endobia*"*	DR	MDR01	113273-113292	338282-338301	20	224989	*pdxJ*/-
		MDR02	113332-113351	338341-338360	20	224989	*pdxJ*/-
		MDR03	138725-138744	138745-138764	20	-	-
		MDR04	141407-141426	141625-141644	20	198	*trnQ*1/*trnQ*2
		MDR05	141448-141467	141666-141685	20	198	*trnQ*1/*trnQ*2
		MDR06	289025-289044	347776-347795	20	58731	*trnF*/*trnA*2
		MDR07	308929-308948	329232-329251	20	20283	-/-
		MDR08	191271-191291	387527-387547	21	196235	*tsf*/-
		MDR09	52452-52473	141300-141321	22	88826	*trnL*2/*trnL*3
		MDR10	171181-171202	231113-231134	22	59910	*pnp*/-
		MDR11	113369-113391	338378-338400	23	224986	*pdxJ*/-
		MDR12	141540-141562	465203-465225	23	323640	*trnM*2/*trnV*2
		MDR13	242541-242563	326616-326638	23	84052	-/*cydD*
		MDR14	308808-308830	329209-329231	23	20378	*tuf*/-
		MDR15	307460-307483	329023-329046	24	21539	*secE*/-
		MDR16	307485-307508	329048-329071	24	21539	*secE*/-
		MDR17	206190-206215	347777-347802	26	141561	*trnA*1/*trnA*2
		MDR18	495524-495551	505578-505605	28	10026	*trnS*3/*trnS*4
		MDR19	307517-307546	329071-329100	30	21524	*secE*/-
		MDR20	130684-130721	132186-132223	38	1464	-/*cysE*
		MDR21	513474-513513	513540-513579	40	26	*acpP*/-
		MDR22	307551-307595	329105-329149	45	21509	*secE*/-
		MDR23	308751-308806	329152-329207	56	20345	*tuf*/-
		MDR24	435418-435492	435493-435567	75	-	-
	IR	MIR01	23640-23659	505488-505507	20	481828	*trnP*1/*trnR*4
		MIR02	141541-141560	347884-347903	20	206323	*trnM*2/*trnI*
		MIR03	208052-208071	289024-289043	20	80952	*trnK*/*trnF*
		MIR04	238440-238459	308844-308863	20	70384	*typA*/*tuf*
		MIR05	347884-347903	465204-465223	20	117300	*trnI*/*trnV*2
		MIR06	133087-133107	133112-133132	21	4	-/-
		MIR07	170354-170374	170535-170555	21	160	*rpsO*/-
		MIR08	389208-389228	505528-505548	21	116299	*trnR*3/*trnR*4
		MIR09	435164-435184	435186-435206	21	1	-/-
		MIR10	459924-459945	516044-516065	22	56098	-/-
		MIR11	81288-81310	81314-81336	23	3	-/-
		MIR12	237863-237886	237897-237920	24	10	*ydjM*/*ydjM*
		MIR13	40549-40588	45537-45576	40	4948	-/-
		MIR14	52338-52380	54429-54471	43	2048	-/-
		MIR15	52376-52435	54375-54434	60	1939	-/-
		MIR16	109083-113105	343701-347723	4023	230595	*

*****Segmental duplications already characterized (Baumann et al., 2002; Lopez-Madrigal et al., 2013a)

Table S3. DNA repeats apparently linked to tRNA genes proliferation. Percentages of each repeat mapping on tRNA stem regions (Stem) or self-paired at the tRNA predicted secondary structure (Self-paired) are indicated.

Repeat	Positions	Sequence	tRNA	Locus	Stem	Self-paired
MDR04	141407-141426<br>141625-141644	TATAGCCAAGCGGTAAGGCA	Gln(TTG)<br>Gln(CTG)	MPC_119<br>MPC_121	40% (8/20)	30% (6/20)
MDR05	141448-141467<br>141666-141685	CCCAGGTTCGAATCCTGGTA	Gln(TTG)<br>Gln(CTG)	MPC_119<br>MPC_121	60% (12/20)	50% (10/20)
MDR06	289025-289044<br>347776-347795	TGCTCTACCAACTGAGCTAT	Phe(GAA)<br>Ala(TGC)	MPC_262<br>MPC_316	45% (9/20)	40% (8/20)
MDR09	52452-52473<br>141300-141321	AGTGGCGAAATTGGTAGACGCA	Leu (CAA)<br>Leu (TAG)	MPC_041<br>MPC_118	36,4% (8/22)	27,3% (6/22)
MDR12	141540-141562<br>465203-465225	CGTAGCTCAGTTGGTTAGAGCAC	Met(CAT)<br>Val (GAC)	MPC_120<br>MPC_418	48% (11/23)	34,8% (8/23)
MDR17	206190-206215<br>347777-347802	GCTCTACCAACTGAGCTATAGCCCCA	Ala(GGC)<br>Ala(TGC)	MPC_175<br>MPC_316	57.7% (15/26)	30,8% (8/26)
MDR18	495524-495551<br>505578-505605	TGAGGGAGGGATTCGAACCCTCGATACA	Ser(GGA)<br>Ser(GCT)	MPC_443<br>MPC_454	71.4% (20/28)	35,7% (10/28)
MIR01	23640-23659<br>505488-505507	GGTCGGAGGTTCGAATCCTC	Pro(GGG)<br>Arg(ACG)	MPC_018<br>MPC_453	45% (9/20)	40% (8/20)
MIR02	141541-141560<br>347884-347903	GTAGCTCAGTTGGTTAGAGC	Met(CAT)<br>Ile(GAT)	MPC_120<br>MPC_317	45% (9/20)	40% (8/20)
MIR03	208052-208071<br>289024-289043	TAGCTCAGTTGGTAGAGCAG	Lys(TTT)<br>Phe(GAA)	MPC_178<br>MPC_262	50% (10/20)	40% (8/20)
MIR05	347884-347903<br>465204-465223	GCTCTAACCAACTGAGCTAC	Ile (GAT)<br>Val(GAC)	MPC_317<br>MPC_418	45% (9/20)	40% (8/20)
MIR08	389208-389228<br>505528-505548	CCGTAGCTCAGCTGGATAGAG	Arg(CCG)<br>Arg(ACG)	MPC_350<br>MPC_453	47.6% (10/21)	28,5% (6/21)
